# commecometrics: an R package for trait-environment modelling at the community level

**DOI:** 10.3897/BDJ.13.e168221

**Published:** 2025-10-16

**Authors:** María A. Hurtado-Materon, Leila Siciliano-Martina, Rachel A. Short, Jenny L. McGuire, A. Michelle Lawing

**Affiliations:** 1 Ecology and Evolutionary Biology Program, Texas A&M University. Department of Ecology and Conservation Biology, Texas A&M University, College Station, United States of America Ecology and Evolutionary Biology Program, Texas A&M University. Department of Ecology and Conservation Biology, Texas A&M University College Station United States of America; 2 Department of Biology, Texas State University, San Marcos, United States of America Department of Biology, Texas State University San Marcos United States of America; 3 Department of Natural Resource Management, South Dakota State University, Rapid City, United States of America Department of Natural Resource Management, South Dakota State University Rapid City United States of America; 4 School of Biological Sciences, Georgia Institute of Technology. School of Earth and Atmospheric Sciences, Georgia Institute of Technology. Interdisciplinary Graduate Program in Quantitative Biosciences., Atlanta, United States of America School of Biological Sciences, Georgia Institute of Technology. School of Earth and Atmospheric Sciences, Georgia Institute of Technology. Interdisciplinary Graduate Program in Quantitative Biosciences. Atlanta United States of America; 5 Department of Ecology and Conservation Biology, Texas A&M University. Ecology and Evolutionary Biology Program, Texas A&M University, College Station, United States of America Department of Ecology and Conservation Biology, Texas A&M University. Ecology and Evolutionary Biology Program, Texas A&M University College Station United States of America

**Keywords:** ecometrics, palaeoecology, community ecology, functional traits.

## Abstract

The R package *commecometrics* provides an accessible, open-access framework for modelling trait–environment relationships using community-level trait data from modern and ancient species. Ecometrics links the trait distributions of communities to their local environmental variables, enabling the reconstruction of past conditions and the prediction of community responses under future climate change. Existing tools for functional trait analysis often lack palaeontological integration or are limited to specific taxa. *commecometrics* addresses these gaps by offering a suite of functions to summarise trait distributions, construct ecometric models, visualise trait–environment relationships, assess model robustness and reconstruct environmental conditions. The package is designed for broad applicability across ecological and palaeoecological studies and includes tools for trait-based biodiversity analysis beyond ecometrics. Through a worked example using carnassial tooth relative blade length (RBL) in carnivoran mammals, we demonstrate the package’s capabilities for analysing trait–environment dynamics across space and time.

## Introduction

Ecometrics is the trait-based quantitative study of the relationship between community-level trait distributions and environmental variables (Polly et al. 2011). The central premise is that certain trait values are more likely to occur in specific environmental settings, allowing the use of community traits to infer local conditions (Polly et al. 2016). Summarising trait data at the community level (e.g. mean, standard deviation) and comparing these summaries with known environmental variables allows ecometric models to infer environmental conditions at fossil sites or predict future trait distributions under climate change (McGuire et al. 2023).

Reconstructing ancient environments is challenging due to the incompleteness of the fossil record, preservation biases and the complexity of geochemical interpretation (Koch 1998, Jackson and Erwin 2006). The latter requires understanding of the chemical processes involved in fossil formation and results are influenced by factors such as diet, physiology and water sources (Koch 1998, Jackson and Erwin 2006). Given the fragmentary and complex nature of the fossil record, multiple lines of evidence and methodological approaches are often necessary to gain a full picture of ancient environments (Jackson and Erwin 2006). Ecometrics offers an alternative framework for reconstructing past environments from fossil remains of ancient communities (Polly and Head 2015). Ecometric models have been applied in mammals (Polly 2010, Short and Lawing 2021, Schap et al. 2024), plants (Dunn et al. 2015, Peppe et al. 2017) and reptiles (Head et al. 2009, Lawing et al. 2012, Parker et al. 2023) to understand the relationship between functional traits and environmental variables such as precipitation, temperature and vegetation cover.

Ecometric analyses have consistently demonstrated strong links between community-level trait distributions and environmental variables. For instance, hypsodonty (an index of tooth crown height to root depth) in mammals reflects annual precipitation experienced by communities of large herbivores (Janis 1988, Fortelius et al. 2002, Eronen et al. 2010). Species in open, arid habitats with grass tend to have higher hypsodonty indices, while those in forested, less abrasive environments have lower values (Eronen et al. 2010). Studies that applied ecometrics to megafaunal communities, revealed that trait compositions reconfigured to align with environmental conditions that underlie massive biodiversity turnover events (Lauer et al. 2023). Other applications have explored the predictive power of functional traits – body size in turtles (Parker et al. 2023), snakes (Lawing et al. 2012) and herbivorous mammals (Lauer et al. 2023, Wilson et al. 2024), diet in herbivorous (Short et al. 2021, Wilson et al. 2024), carnivorous (Siciliano‐Martina et al. 2024) and small mammals (Schap et al. 2024) and locomotion traits in carnivores (Polly 2010) and artiodactyls (Short and Lawing 2021, Short et al. 2023) – demonstrating that ecometric models can capture environmental patterns at regional and continental scales and can provide informative palaeoclimate reconstructions. These results highlight the value of ecometrics as a functional, comparative tool to model biotic responses to changes in climate and to infer past environmental conditions. Despite their utility, ecometrics methods have been limited by the absence of an accessible, standardised computational platform.

R packages for analysing functional traits have been developed in recent years. Current packages focus on trait manipulation, the estimation of functional spaces based on traits (Carmona et al. 2024) and the access to and exploration of databases containing functional traits and environmental variables (Denelle et al. 2023, Lam et al. 2024). Some also summarise functional trait distributions at the community level (Pavoine 2020, Zhang et al. 2021, Magneville et al. 2022, Grenié and Gruson 2023) and enable the analysis of ecological data from a metacommunity perspective (Debastiani and Pillar 2012). However, these packages are primarily focused on plants, lack easy integration with geographic space and do not include functions to reconstruct palaeoenvironments based on the fossil record.

To address these gaps, we introduce *commecometrics*, an R package that facilitates the integration of trait data with environmental predictors across temporal and spatial scales. It offers a workflow for summarising community trait metrics, building ecometric models and evaluating trait–environment relationships with visualisation tools. In this paper, we describe the structure and functionality of *commecometrics*, demonstrate its use with an empirical dataset and discuss potential applications in conservation and palaeoecology. *commecometrics* is intended for ecologists, palaeontologists and conservation biologists interested in using trait-based methods to explore how traits within communities reflect their environments in the past or present. The *commecometrics* package is modular, allowing users to apply individual components of the workflow independently, such as summarising traits, testing models, or reconstructing past climates, depending on the structure of their data and research goals.

## Installation

The *commecometrics* package is available on CRAN and can be installed using the standard *install.packages()* function. The development version is hosted on GitHub.

# Install the stable version from CRAN

install.packages("commecometrics")

# Install the development version from GitHub

# First install devtools if not already installed

install.packages("devtools")

devtools::install_github("mariahm1995/commecometrics")

## Usage

**Figure 1.**
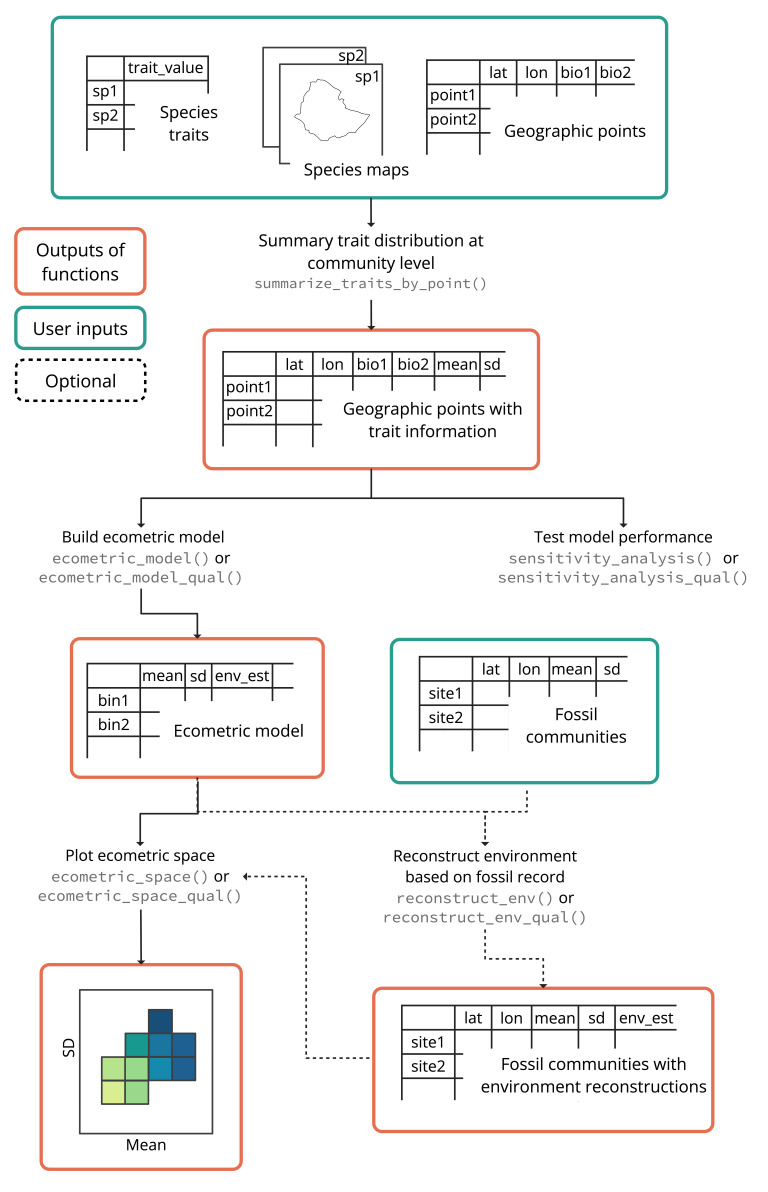
Workflow of the *commecometrics* R package. The package implements a modular pipeline to model and reconstruct trait–environment relationships. Green boxes represent user-provided inputs, including modern and fossil species-level trait data, species distributions and environmental sampling points. Red boxes indicate outputs generated by package functions. Dashed lines represent optional steps. Community-level trait summaries (e.g. mean, standard deviation) are calculated using *summarize_traits_by_point()* and can be modelled against environmental variables using *ecometric_model()* or *ecometric_model_qual()* for continuous or categorical traits, respectively. Model performance can be assessed with sensitivity analyses and trait–environment patterns can be visualised using ecometric space plots. Finally, ecometric models can be applied to fossil trait data to reconstruct past environmental conditions.

The *commecometrics* package provides a modular workflow for analysing trait–environment relationships at the community level and reconstructing past environments using fossil trait data. Fig. 1 outlines the main components of the workflow.

The process begins with three core inputs: (1) a dataset with species-level trait values; (2) a set of species distribution maps in shapefile format and (3) a set of geographic sampling points with associated environmental variables. These inputs are combined using the *summarize_traits_by_point()* function, which calculates community-level trait distributions (e.g. mean and standard deviation) at each geographic point based on the species present. These summarised trait data form the basis for all downstream analyses, but may be a desired output in itself.

After the first step, users can: (1) Build an ecometric model using *ecometric_model()* or *ecometric_model_qual()* (for categorical traits). These models describe how trait distributions relate to environmental variables across the landscape; (2) Evaluate model performance using *sensitivity_analysis()* or *sensitivity_analysis_qual()* to test robustness and assess how well the model captures the underlying trait–environment relationships; (3) Visualise the ecometric space using *ecometric_space()* or *ecometric_space_qual()*, which plots environmental estimates across trait value bins, providing an intuitive map of how traits vary with environment and (4) Reconstruct environmental conditions at fossil sites using *reconstruct_env()* or *reconstruct_env_qual()*. These functions estimate past environmental conditions based on the trait composition of fossil communities.

*commecometrics* provides a flexible and modular framework for conducting ecometric analyses. While outputs from one function (e.g. community-level trait summaries) can be passed to subsequent functions (e.g. model fitting), users are not required to follow a rigid pipeline. For instance, the arguments *comm_metric_1* and *comm_metric_2* in the *summarize_traits_by_point()* function allow users to apply any summary function to community trait data. However, users may supply a custom dataframe, such as one containing community-weighted trait means based on energy intake and use it directly in downstream modelling functions (Žliobaitė and Lawing 2025). This flexible design supports a wide range of applications.

## Usage Beyond Ecometric Analysis

The R package commecometrics supports a wide range of community-level analyses beyond ecometric applications (see Table 1 for function descriptions). It allows users to compile species lists for specific geographic locations, calculate trait-based metrics and assign communities to continents based on spatial coordinates. These capabilities are broadly applicable to studies examining various dimensions of biodiversity. In particular, the function *summarize_traits_by_point()* can be employed in diverse analytical contexts beyond traditional ecometric modelling. Trait distributions are calculated for each community using a user-specified function. By default, the mean and standard deviation are computed, as these are commonly used in ecometric analyses. However, users can substitute any function that accepts a numeric vector as input, including the fd_ functions from the R package fundiversity (see example below), to derive other functional diversity metrics (Grenié and Gruson 2023).

traitsByPoint <- summarize_traits_by_point(

points_df = geoPoints,

trait_df = traits,

species_polygons = spRanges,

summary_trait_1 = function(x) fundiversity::fd_fdis(x),

trait_column = "RBL",

species_name_col = "sci_name",

continent = TRUE,

parallel = TRUE)

**Figure 2.**
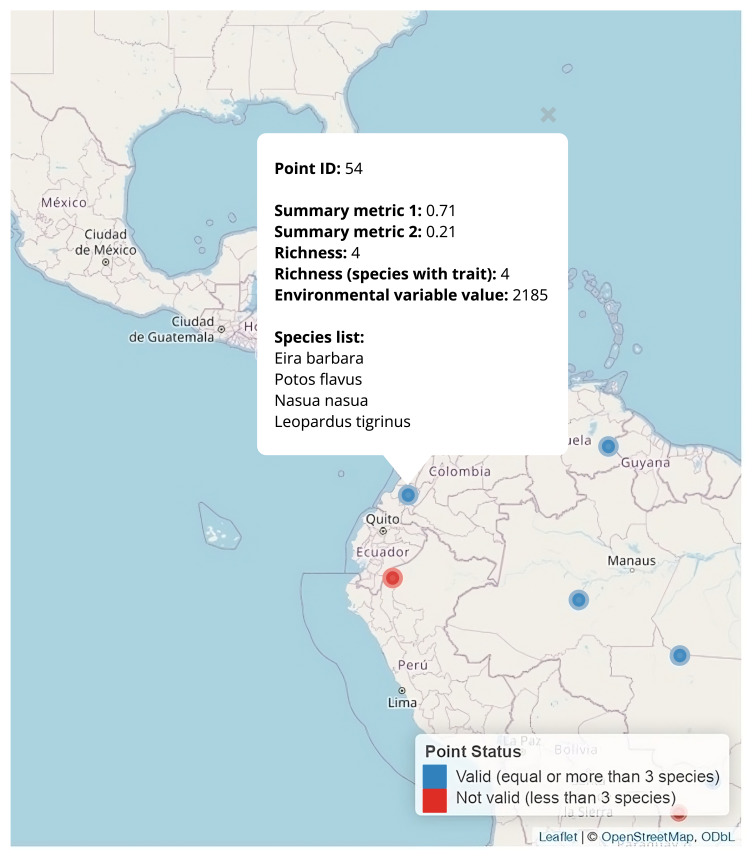
Interactive map output from the *inspect_point_species()* function, displaying species composition and associated metrics for each sampling point. Pop-up windows provide information per point, including species identity, summary trait metrics, species richness, richness based on trait availability and the value of an environmental variable. Point status is colour-coded: blue indicates valid points (≥ 3 species) and red indicates non-valid points (< 3 species) based on the species richness threshold.

The function *inspect_point_species()* allows users to verify the species present at each geographic point and review associated metrics. It supports data exploration by providing a visual, interactive interface for examining large datasets. Using the *leaflet* package (Cheng et al. 2015), the function generates an interactive map that displays selected sampling points as coloured markers: blue for points with enough species that have trait data (above a user-defined threshold) and red for those that fall below. Clicking on any point opens a pop-up showing the point ID, trait summary statistics (mean, standard deviation, richness), optional environmental variable values and a list of overlapping species (Fig. 2). This tool facilitates interpretability by linking spatial locations to ecological and trait-based summaries in an intuitive format, making it broadly applicable to ecological and biogeographic research.

## Example

To demonstrate the functionality of *commecometrics*, we applied the package to a dataset of carnivoran carnassial tooth relative blade length (RBL) to test its relationship with the habitat structure (Siciliano‐Martina et al. 2024). This example illustrates the complete workflow: summarising trait data, fitting a categorical ecometric model, projecting fossil sites, visualising ecometric space and evaluating model performance.

Step 1: Load and prepare data

This example uses three input datasets provided by the user. The file sampling_points.csv contains environmental sampling locations and includes a column named "VegSimple", which classifies each point into a categorical vegetation type. The file traits.csv provides species-level values for the carnassial tooth relative blade length. Finally, species distribution data are provided in a shapefile containing geographic range polygons for all terrestrial mammals. In this analysis, the dataset is filtered to include only species belonging to the order Carnivora. The polygons were downloaded from the IUCN Red List website, which hosts expert-reviewed range maps for many taxonomic groups (IUCN 2025). Any taxonomic group with available distribution data on the IUCN platform can be used with the commecometrics package.

# Download data from Figshare

options(timeout = 600)

download.file("https://ndownloader.figshare.com/files/56228033", destfile = "data.zip", mode = "wb")

unzip("data.zip")

# Load data

points <- read.csv("data/sampling_points.csv")

traits <- read.csv("data/traits.csv")

fossil <- read.csv("data/fossil_RBL.csv", header = TRUE)

geometry <- sf::st_read("data/data_0.shp")

geography$SCI_NAME <- gsub(" ", "_", geography$SCI_NAME)

Step 2: Summarise traits at sampling points

The first step is to calculate community-level trait summaries at each environmental sampling point. This is done by intersecting each point with the species range polygons to determine which species are present and then computing the mean and standard deviation of the selected trait across those species. This summarised trait information forms the basis for all downstream ecometric modelling steps.

traitsByPoint <- summarize_traits_by_point(

points_df = points,

trait_df = traits,

species_polygons = geometry,

species_name_col = "SCI_NAME",

trait_column = "RBL")

To explore the species composition at sampling points, users can interact with the *inspect_point_species()* function, described in the Usage Beyond Ecometric Analysis section. If *point_ids* argument is left NULL, the function selects a random sample of 10 points. Otherwise, users can inspect specific point IDs to examine known or targeted locations. The user can also select the number of points to inspect via the argument *n_random*.

# View species present at randomly selected points with at least 3 species with trait data

inspect_point_species(

traits_summary = traitsByPoint,

min_species_valid = 3)

# View species at specific point IDs

inspect_point_species(

traits_summary = traitsByPoint,

min_species_valid = 3,

point_ids = c("113435", "99936", "101328"),

ID_col = "GlobalID")

Step 3: Build a categorical ecometric model

With community-level trait summaries available, the next step is to build an ecometric model that relates trait distributions to a categorical environmental variable. In this case, we use "VegSimple", a simplified vegetation classification. The model bins the trait space (mean and standard deviation of RBL) into a grid and assigns each bin to the most likely vegetation category based on the observed data. Predicted environmental variables are calculated using Maximum Likelihood; this method has been shown to produce the most accurate estimates from community trait values (Short et al. 2021).

modelVeg <- ecometric_model_qual(

points_df = traitsByPoint$points,

category_col = "VegSimple",

grid_bins_1 = 25,

grid_bins_2 = 25)

If users do not specify the number of bins, the function automatically determines an optimal bin number using *optimal_bins()*, which implements Scott’s rule (Scott 1979, Lauer et al. 2023). This approach selects bin widths based on data spread and sample size, aiming to balance detail and generalisation. The commecometrics package includes the *optimal_bins()* function to help users assess and set appropriate bin numbers for their datasets, ensuring that the model captures meaningful structure in the trait–environment relationship without overfitting or excessive generalisation.

Step 4: Reconstruct environments at fossil sites

Once an ecometric model has been fitted, it can be used to estimate the most likely environmental category at fossil sites based on the trait composition of fossil communities. Here, we use a fossil dataset with RBL summaries and reconstruct the corresponding vegetation category. This function assigns fossil communities to the most probable vegetation category based on their trait values. If match_nearest = TRUE, the function also assigns each fossil site to its nearest modern sampling point.

# Reconstruct vegetation category at fossil sites using the categorical ecometric model

fossil_results <- reconstruct_env_qual(

fossildata = fossil,

model_out = modelVeg,

match_nearest = TRUE,

fossil_lon = "long",

fossil_lat = "lat",

modern_id = "GlobalID",

modern_lon = "Longitude",

modern_lat = "Latitude")

Step 5: Visualise the ecometric space

**Figure 3.**
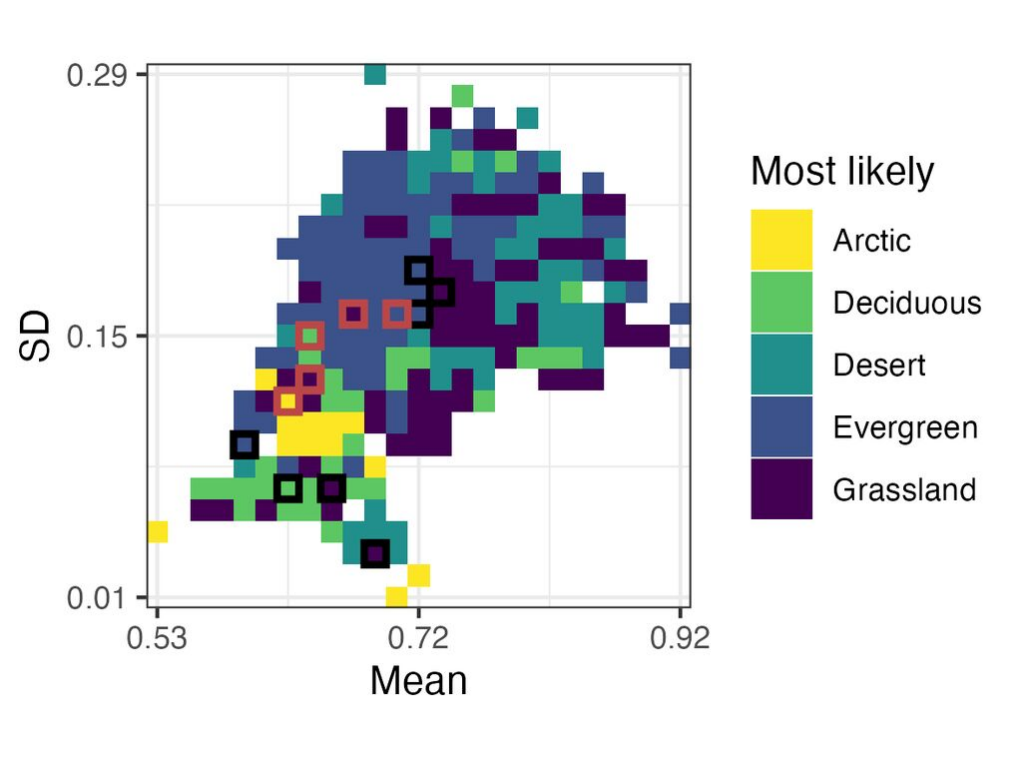
Predicted ecometric space for vegetation categories. Each grid cell represents a bin in the trait space defined by the mean and standard deviation (SD) of the RBL, summarised at the community level. Colours indicate the most probable vegetation category predicted by the ecometric model. Black and red outlines mark fossil and modern trait bins, respectively, allowing direct comparison between past and present community trait compositions.

The ecometric model can be visualised using the *ecometric_space_qual()* function, which generates two types of outputs. The first is the category map (Fig. 3), which shows the most probable environmental category (e.g. vegetation type) predicted for each combination of community-level mean (x-axis) and standard deviation (y-axis) trait values (the ecometric trait space). Each grid cell represents a bin in this space and its fill colour corresponds to the predicted category. If fossil data are provided via the fossil_data argument, the corresponding fossil and modern trait bins are outlined: fossil communities are shown with black outlines and their nearest modern analogues are shown with red outlines (by default). This plot is useful for identifying distinct trait–environment associations, visualising community structure and verifying how fossil communities map on to modern ecometric space. Since *ecometric_space_qual()* returns a ggplot2 object, it can be saved using the *ggsave()* function from the ggplot2 package (Wickham et al. 2007).

plots <- ecometric_space_qual(

model_out = modelVeg,

fossil_data = fossil_results,

x_label = "Mean",

y_label = "SD")

library(ggplot2)

ggsave("ecometric_space.png", plots[[1]],

width = 10, height = 7.5, units = "cm")

**Figure 4.**
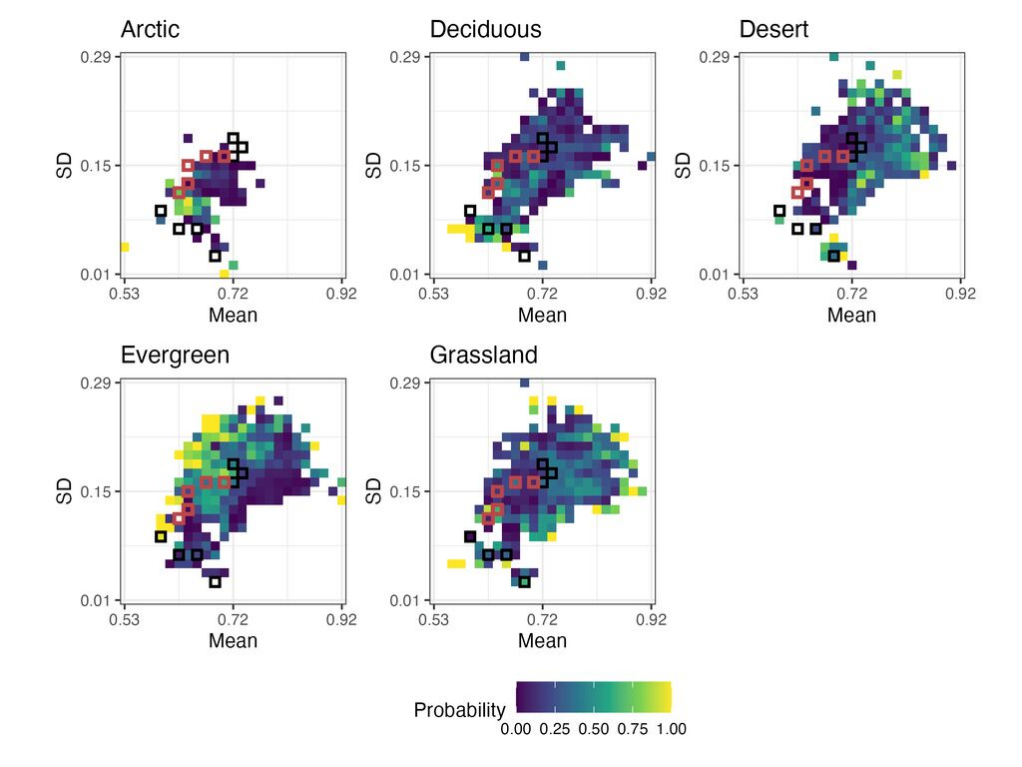
Probability maps for each vegetation category across ecometric trait space. Each panel shows the probability that a given trait bin (defined by community-level mean and standard deviation of RBL corresponds to a specific vegetation category: arctic, deciduous, desert, evergreen or grassland. Warmer colours indicate higher probabilities, while bins with zero probability are rendered transparent. Red outlines mark the trait bins of fossil communities and black outlines indicate their nearest modern analogues in trait space

The second output is a set of probability maps (Fig. 4), with one map generated for each environmental category. These plots show the probability that each trait bin belongs to a given category. The colour gradient indicates the probability (from low to high) that a particular bin is associated with that environment, while bins with zero probability are rendered transparent to highlight informative areas of trait space. If fossil_data is included, fossil and modern analogue bins are outlined as in the category map. These probability maps help users evaluate areas of overlap or uncertainty and determine how confidently trait combinations can be assigned to specific categories. To display all maps together, individual ggplot2 plots are combined using the patchwork package (Pedersen 2019), which simplifies the layout of complex multi-panel figures.

library(patchwork)

combinePlot <- plots[[2]][[1]] + plots[[2]][[2]] + plots[[2]][[3]] +

plots[[2]][[4]] + plots[[2]][[5]] + plot_layout(guides = "collect") &

theme(legend.position = "bottom")

ggsave("probability_maps.png", combinePlot,

width = 20, height = 15, units = "cm")

Step 6: Assess model sensitivity

Finally, the robustness of the ecometric model can be evaluated using *sensitivity_analysis_qual()*. This function performs a bootstrap resampling procedure to test how the model performs under varying sample sizes. It provides both training accuracy (sensitivity) and test accuracy (transferability), which are critical for understanding model limitations.

sensitivity_results <- sensitivity_analysis_qual(

points_df = traitsByPoint$points,

category_col = "VegSimple",

sample_sizes = seq(100,10000,1000))

This example demonstrates how *commecometrics* can be applied to model the relationship between functional traits and categorical environmental variables and to reconstruct palaeoenvironments from fossil data. The same workflow can be applied to continuous environmental variables using the parallel functions *ecometric_model()*, *ecometric_space()* and *reconstruct_env()*.

## Limitations

The utility of *commecometrics* is currently constrained by data availability. The package is designed for taxonomic groups for which both species distribution and functional trait databases exist, most commonly plants and vertebrates (Vermillion et al. 2018). As a result, its applicability to many other groups remains limited due to the relative scarcity of trait and range datasets. Additionally, the palaeoecological applications of *commecometrics* require that traits be reliably measurable from the fossil record. This restricts trait selection to features that can be measured throughout fossilised material (e.g. dental or skeletal), which may not be feasible for all taxa or traits of interest.

We emphasize the importance of continued efforts to collect and share data for undersampled taxonomic groups. Expanding trait–environment frameworks like ecometrics to a broader range of organisms will provide a more comprehensive view of ecological dynamics and improve our understanding of ecosystem responses to climate change (Short et al. 2023). We invite researchers and data providers to contribute to this goal, thereby enhancing our collective ability to study biodiversity patterns across spatial and temporal scales.

## Conclusion

It is critical that we understand how communities have and will respond to changing environmental conditions. The *commecometrics* package provides many tools to facilitate these analyses. At its full capability, it can be used to build ecometric models, which establish trait-environmental relationships and reconstruct past, present and future environmental conditions given community compositions.

Component functions are even more helpful to a broader suite of biodiversity analyses. Ecometric models provide a means to quantitatively assess past environmental conditions, allowing us to determine how ecosystems have shifted in response to fine-scale change. These analyses will be crucial for evaluating which modern plant and animal communities will need to reconfigure in response to ongoing global change and, potentially, for evaluating how and where those reconfigurations could occur given modern species distributions.

## Figures and Tables

**Table 1.** Functions included in the R package commecometrics, with brief descriptions of their purpose. For more detailed information, consult the package manual or use the help documentation for individual functions.

**Function name**	**Description**
**Functions for general community analysis**	
summarize_traits_by_point()	Provides two trait-based metrics for each geographic point.
inspect_point_species()	Creates an interactive map to verify species overlap and information related to selected points.
optimal_bins()	Calculates the optimal number of bins for a numeric vector based on Scott's rule.
**Functions exclusive to ecometric analysis**	
ecometric_model() and ecometric_model_qual()	Builds an ecometric trait space for quantitative and qualitative environmental variables.
ecometric_space() and ecometric_space_qual()	Visualises the ecometric space.
reconstruct_env() and reconstruct_env_qual()	Uses fossil community trait summaries to reconstruct past environmental conditions.
sensitivity_analysis() and sensitivity_analysis_qual()	Evaluates the performance of ecometric models.
